# A novel non-linear approach for establishing a QSAR model of a class of 2-Phenyl-3-(pyridin-2-yl) thiazolidin-4-one derivatives

**DOI:** 10.3389/fphar.2023.1263933

**Published:** 2023-09-27

**Authors:** Leilei Wu, Yonglin Chen, Kangying Duan

**Affiliations:** Spine Surgery, Affiliated Hospital of Weifang Medical University, Weifang, Shandong, China

**Keywords:** osteosarcoma, QSAR, gene expression programming, model, non-linear

## Abstract

In this investigation, we aimed to address the pressing challenge of treating osteosarcoma, a prevalent and difficult-to-treat form of cancer. To achieve this, we developed a quantitative structure-activity relationship (QSAR) model focused on a specific class of compounds called 2-Phenyl-3-(pyridin-2-yl) thiazolidin-4-one derivatives. A set of 39 compounds was thoroughly examined, with 31 compounds assigned to the training set and 8 compounds allocated to the test set randomly. The goal was to predict the IC_50_ value of these compounds accurately. To optimize the compounds and construct predictive models, we employed a heuristic method of the CODESSA program. In addition to a linear model using four carefully selected descriptors, we also developed a nonlinear model using the gene expression programming method. The heuristic method resulted in correlation coefficients (*R* ^2^) of 0.603, 0.482, and 0.107 for R^2^_cv_ and S^2^, respectively. On the other hand, the gene expression programming method achieved higher *R* ^2^ and S^2^ values of 0.839 and 0.037 in the training set, and 0.760 and 0.157 in the test set, respectively. Both methods demonstrated excellent predictive performance, but the gene expression programming method exhibited greater consistency with experimental values. The successful nonlinear model generated through gene expression programming shows promising potential for designing targeted drugs to combat osteosarcoma effectively. This approach offers a valuable tool for optimizing compound selection and guiding future drug discovery efforts in the battle against osteosarcoma.

## 1 Introduction

Osteosarcoma is a malignant tumor that arises from mesenchymal tissue and is characterized by the direct production of bone or osteoid tissue by rapidly proliferating tumor cells (Ghashghaeinia et al., 2019; Yang et al., 2023). It is the most common primary malignant bone tumor and exhibits a high degree of malignancy, rapid growth, and early metastasis. Unfortunately, early diagnosis of osteosarcoma is challenging, and the prognosis is generally poor. The statistical incidence is estimated to be around 4–5 cases per 1 million individuals. Osteosarcoma primarily affects individuals in their teenage years, with the average median age of diagnosis being 15 years. The highest occurrence is observed between the ages of 15 and 20, with 60% of cases occurring in individuals below the age of 25. Patients over the age of 40 with osteosarcoma are commonly associated with Paget’s disease of bone or have a history of radiotherapy. Conventional osteosarcomas, known as classic osteosarcomas, predominantly originate in the bone marrow and account for approximately 80% of all osteosarcoma cases. They are further classified into sub-types including osteogenic (50%), chondrogenic (25%), and fibrogenic (25%). Additionally, there are rare subtypes of osteosarcoma such as capillary dilatation, small cell, parabone, periosteal, highly malignant surface osteosarcoma, low malignant central osteosarcoma, multicenter osteosarcoma, and secondary osteosarcoma (associated with Paget’s disease). Traditional osteosarcomas mainly occur in the long bones of the extremities, particularly around the knee joint, such as the distal femur and proximal tibia. Approximately 91% of cases are located in the metaphysis (near the growth plate), while 9% occur in the diaphysis (shaft of the bone). Atypical osteosarcomas can also affect non-long bones such as the skull, pelvis, mandible, and vertebrae, and their incidence tends to increase with age. The common initial symptoms of osteosarcoma include pain, swelling, the presence of a painful mass, and inflammatory reactions. As the disease progresses, there may be varying degrees of joint movement limitation and the occurrence of pathological fractures.

Currently, the standard treatment approach for osteosarcoma involves a combination of preoperative neoadjuvant chemotherapy, surgical resection, and postoperative adjuvant chemotherapy. The primary first-line chemotherapy drugs utilized for osteosarcoma include methotrexate (MTX) (Azadian et al., 2023), doxorubicin (ADM) (Hua et al., 2023), cisplatin (DDP) (Kucan et al., 2023), ifosfamide (IFO) (Almesned et al., 2023), vincristine (VCR), epirubicin (EPI) (Esparragosa Vazquez et al., 2023), cyclophosphamide (CTX) (Zhang et al., 2023), and etoposide (VP-16) (Wang et al., 2010), among others. Among these, MTX, ADM, DDP, and IFO are the most commonly employed drugs. However, in the clinical practice of osteosarcoma chemotherapy, it has been observed that these drugs often come with significant side effects. For instance, methotrexate and cisplatin can lead to kidney damage, doxorubicin can cause cardiac inhibition, and chemotherapy drug resistance is a common issue. Therefore, there is an urgent need to develop and design new, more effective drugs specifically for osteosarcoma that can overcome these limitations and minimize adverse effects.

*In vitro* studies using osteosarcoma cell lines have demonstrated the cytotoxic effects of 2-Phenyl-3-(pyridin-2-yl) thiazolidin-4-one derivatives (Deng et al., 2022). These compounds have shown potent antiproliferative activity, promoting apoptotic cell death and inhibiting tumor cell migration and invasion. Furthermore, *in vivo* studies utilizing osteosarcoma xenograft models have shown promising results, with reduced tumor growth and improved survival rates following treatment with these derivatives.

The potential advantages of 2-Phenyl-3-(pyridin-2-yl) thiazolidin-4-one derivatives for osteosarcoma therapy include their ability to target multiple pathways involved in tumor progression and their favorable toxicity profiles compared to conventional chemotherapeutic agents. However, challenges remain in terms of optimizing their pharmacokinetic properties, understanding their precise molecular targets, and investigating potential drug resistance mechanisms.

Quantitative structure-activity relationship (QSAR) (Feng et al., 2023; Karaduman and Kelleci Celik, 2023) is a method of drug research that uses mathematical models to describe the relationship between molecular structure and certain biological activities of molecules. This method has been widely used in drug activity prediction and the development of new drugs. 2D QSAR is a computational approach used in drug discovery to establish a relationship between the chemical structure of a molecule and its biological activity. It involves the analysis of molecular descriptors, which are numerical representations of various structural and physicochemical properties of molecules, and their correlation with the observed biological activities or properties. In 2D QSAR, the focus is primarily on two-dimensional representations of molecules, such as molecular graphs or simplified molecular input line entry system (SMILES) notations. By focusing on two-dimensional molecular descriptors such as logP, molecular weight, and various counts of atoms and functional groups, 2D QSAR provides a computationally efficient approach that does not require the three-dimensional structure of the molecules. This makes it especially useful for handling large datasets and exploring vast chemical spaces. Additionally, the simplicity of 2D QSAR allows for easier interpretation and visualization of the relationships between structural features and activity, aiding in the understanding of underlying mechanisms. While it might not capture the full complexity of molecular interactions, the speed, simplicity, and interpretability of 2D QSAR make it a valuable tool in early-stage drug discovery, virtual screening, and prioritization of compounds for further experimental analysis. This 2D QSAR model will help for further new 2-Phenyl-3-(pyridin-2-yl) thiazolidin-4-one derivatives finding. To found the more prediction QSAR model, non-linear method was used in our work.

Gene Expression Programming (GEP) (Ipek et al., 2022) stands out as a nonlinear approach for QSAR modeling, offering significant advantages. Its automated feature generation capability unveils intricate descriptor-activity relationships often overlooked by manual selection. GEP adeptly captures nonlinear interactions among descriptors, enabling the modeling of complex molecular behaviors. This method’s adaptability to noisy data ensures robustness in handling complex biological responses. Additionally, GEP’s capacity to incorporate domain knowledge and apply regularization reduces overfitting risks, enhancing predictive accuracy. Furthermore, the interpretability of the evolved expressions provides insights into molecular mechanisms. GEP’s versatility, scalability, and potential for scientific discovery make it a powerful choice for QSAR modeling, addressing the limitations of linear methods and offering a comprehensive tool for understanding complex molecular activities.

## 2 Methods

The study referenced in literature involved a dataset of 39 compounds, along with their corresponding IC_50_ values (Deng et al., 2022). To simplify the analysis, the logarithm of these values was calculated. The structures of all the compounds, along with their log (IC_50_) values and the predicted values, are presented in Table 1. To assess the predictive performance of the model, the dataset was randomly divided into two subsets: a training set comprising 31 structures used for constructing the model, and a test set containing 8 structures used to evaluate the model’s prediction capabilities. This division allowed for the validation of the model’s effectiveness in predicting the IC_50_ values of unseen compounds.

**TABLE 1:** Experimental and calculated log(IC_50_) of 28 compounds (HM and GEP).

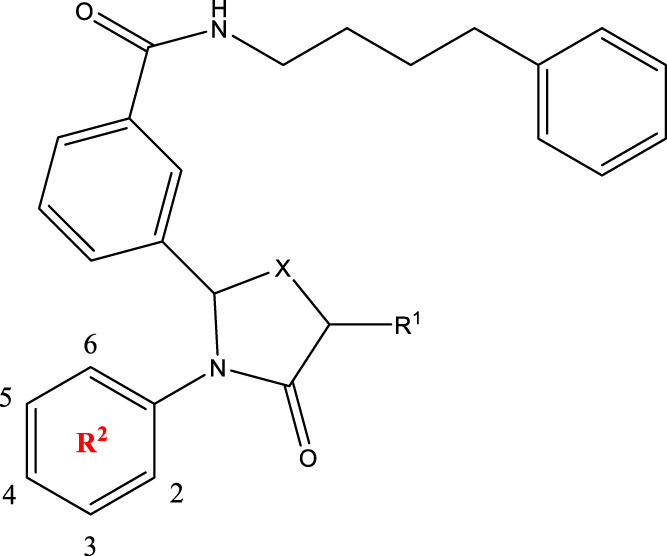 2a-o					
Compd	R2	R1	X	IC_50_(nM)	
2a	3-F	H	S	32.4	
2b	2-F	H	S	34.1	
2c	3-CF3	H	S	29.1	
2d	H	H	S	15.9	
2e	3-CH3	H	S	93.2	
2f	2-OCH3	CH3	S	45.1	
2g	2-OC2H5	CH3	S	71.6	
2 h	3-F-2-OCH3	H	S	26.2	
2i	5-F-2-OCH3	H	S	71.1	
2j	2,4-Di-OCH3	H	S	8.8	
2 k	2-OCH3-5-CH3	H	S	90.4	
2L	2-OCH3-5-CH3	H	S	300.2	
2o	3-F	H	O	319.6	
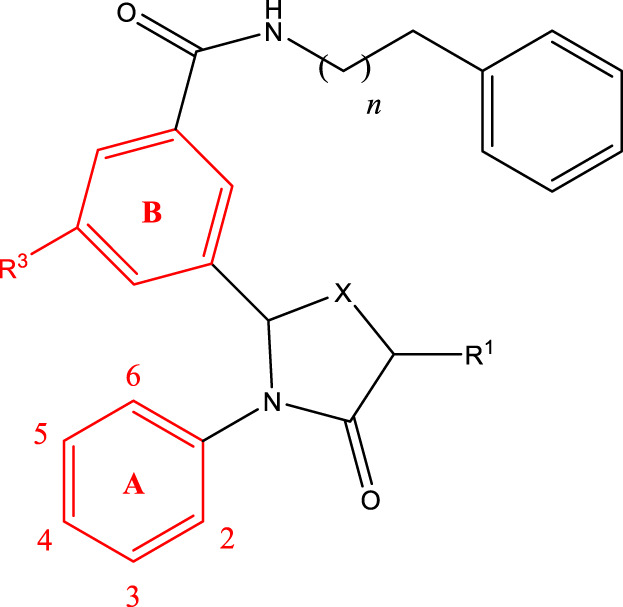 5a-f					
Compd.	R2	R3	R1	n	IC_50_(nM)
5b	3-cyclopropyl	H	H	3	298.8
5c	4-cyclopropyl	H	H	3	217.1
5f	3-ethynyl	H	H	3	149.2
5g	3-ethynyl-5-OCH3	H	H	1	291.6
5 h	2-OCH3	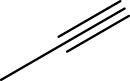	H	2	40.8
5i	2-OCH3	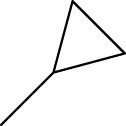	H	3	23.5
5j	2-OCH3	F	H	3	40.9
5 k	2-OCH3	CH3	H	3	16.2
5L	4-cyclopropyl	H	CH3	3	191.3
5 m	3-cyclopropyl	H	CH3	3	294.1
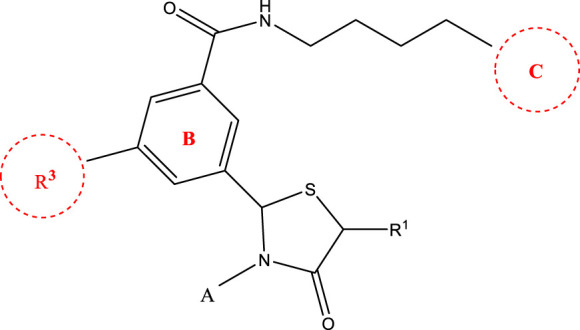 6a-h,7a-k,8a-c					
Compd.	A	R3	R1	C	IC_50_(nM)
6c	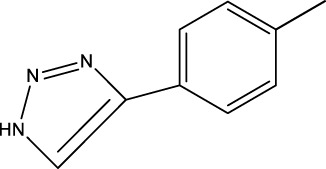	H	H	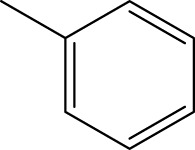	109.7
6g	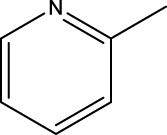	H	H	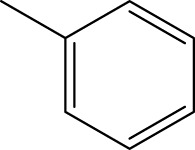	19.7
6 h	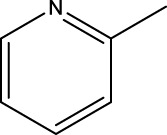	H	H	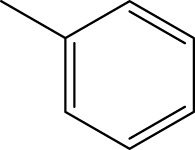	14.2
7a	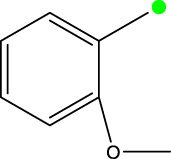	H		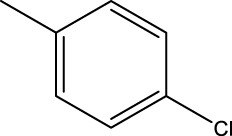	78.6
7c	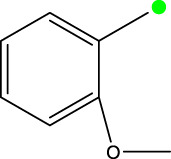	H	CH3	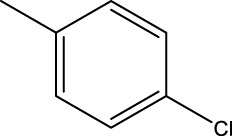	931.9
7e	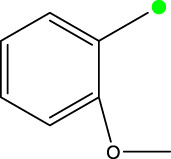	H	H	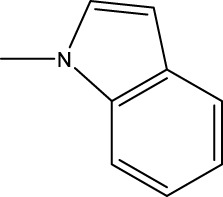	285.5
7f	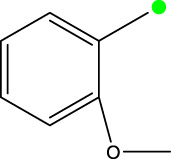	H	H	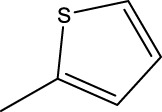	46.0
7i	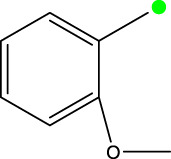	H	H	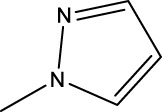	91.7
7j	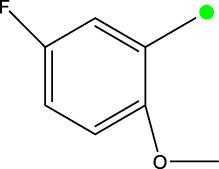	H	H	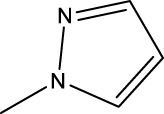	97.3
7 k	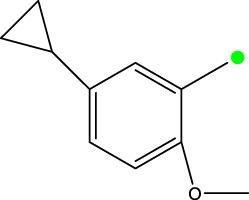	H	H	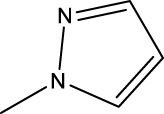	298.5
8b	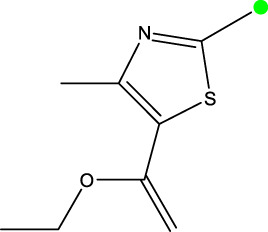	H	H	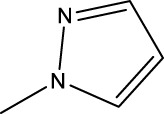	429.5
8c	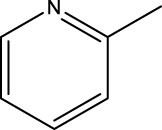	H	H	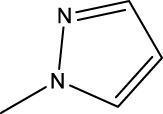	60.3
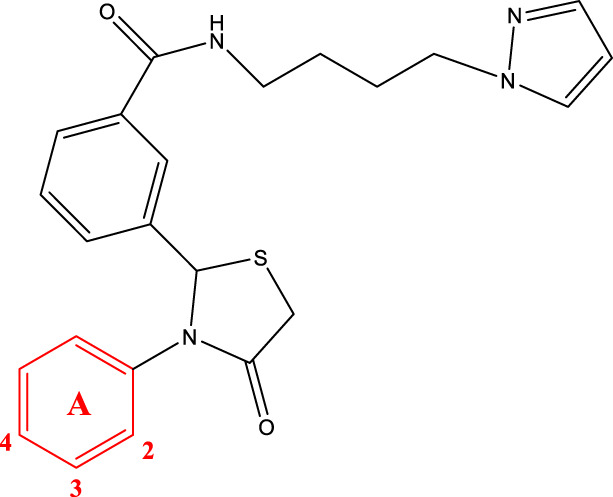 8f-i					
Compd.	A	IC_50_(nM)			
8f	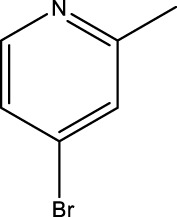	105.4			
8g	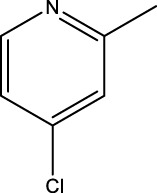	36.4			
8 h	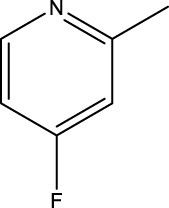	33.2			
8i	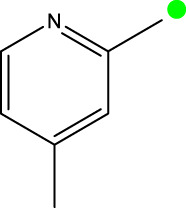	19.6			

The initial step involved plotting the structures of the 39 compounds using ChemDraw software. Subsequently, these structures were imported into HyperChem software (Froimowitz, 1993) for geometry optimization, employing the MM + molecular mechanical force field and semi-empirical AM1 approaches. The optimization process aimed to refine the molecular geometry and ensure its stability. Afterwards, the MOPAC6.0 (Gieseking, 2021) program was utilized to generate the. arc, .end, and. mno files for each compound. These files contain important information about the molecular properties and characteristics. To further analyze and predict the properties of the compounds, the exported files were then submitted to the CODESSA software (Katritzky et al., 2006). This software allowed for the computation and classification of various molecular descriptors, which were categorized into three classes: electrostatic, topological, and quantum mechanical descriptors. These descriptors provide numerical information about specific features of the molecular structure. By establishing an equation based on these molecular descriptors, subsequent predictions could be made. The derived equation allowed for the correlation between the chemical structure and the desired properties, facilitating the prediction of important characteristics for the compounds under investigation.

### 2.1 The linear model by heuristic method

The heuristic method employed an optimal multiple linear regression approach to create a linear model, establishing correlations between the descriptors calculated by CODESSA and the biological activity of the compounds. The primary objective was to pinpoint the descriptor that best represents the relationship between the chemical structure and biological activity. To gauge the model’s accuracy, several statistical parameters were utilized. The regression coefficient (*R* ^2^) was employed to evaluate the goodness of fit, indicating the proportion of variation in the biological activity that can be explained by the model. Cross-validation regression coefficients (*R* ^2^) were used to assess the model’s performance on unseen data, providing an estimate of its predictive ability. Additionally, the standard deviation (S^2^) was calculated to estimate the data dispersion around the fitted line. The linear model generated by the heuristic method involved four descriptors, which are detailed in Table 2. Descriptor selection processes using heuristic algorithms typically involve the following steps: Start with a pool of available descriptors that can be calculated for the given dataset of chemical compounds. Define a scoring function or criterion that quantifies the relevance or importance of each descriptor in relation to the property or behavior you are trying to predict or analyze. Begin with an initial subset of descriptors. This subset can be chosen randomly, using domain knowledge, or through other methods. Use a heuristic algorithm, such as a genetic algorithm, simulated annealing, or particle swarm optimization, to iteratively refine the descriptor subset. This involves selecting a subset of descriptors, evaluating their performance using the scoring function, and then modifying the subset to improve the score. If using a genetic algorithm, employ crossover and mutation operations to create new descriptor combinations based on the existing ones. This introduces diversity and explores different combinations. Evaluation: Calculate the fitness or score of each descriptor subset using the defined scoring function. This indicates how well the selected descriptors predict the property of interest. Based on the fitness scores, select promising descriptor subsets for the next iteration. Heuristic algorithms often favor subsets with higher scores, but they might also allow some lower-scoring subsets to maintain diversity and explore different options. Define stopping conditions, such as a maximum number of iterations, reaching a desired level of performance, or observing diminishing improvements. Once the algorithm terminates, the final selected descriptor subset is obtained. This subset is expected to contain a limited number of relevant descriptors that effectively capture the underlying patterns in the data. These descriptors were selected based on their strong correlation with the biological activity, as determined by the multiple linear regression analysis.

**TABLE 2:** Selected descriptors and statistical parameters.

Descriptor	Physical-chemical meaning	Coefficient	T-test
Constant	—	−9.023	3.528
MSA	Molecular surface area	−1.095e-03	4.136
ANRIFN	Avg nucleoph. react. index for a N atom	8.299	4.278
FFPQC	FPSA-2 Fractional PPSA (PPSA-2/TMSA) [Quantum-Chemical PC]	6.249e-02	3.551
TM2RE	Tot molecular 2-center resonance energy	4.720	2.719

### 2.2 The nonlinear model by gene expression programming

GEP is a computational method that incorporates five essential elements (Lakshmi et al., 2022). The first element is the termination condition, which determines the stopping criterion for the algorithm’s search for solutions. It defines when the algorithm should halt its iterative process.

The second element is the fitness function, which assesses and quantifies the quality or performance of each individual in the population. The fitness function serves as the evaluation metric for determining the reproductive success of individuals.

The third element is the functional set, which consists of mathematical functions like subtraction (−), division (/), and a set of mathematical constants (Q). These functions and constants are used in constructing expression trees, which are a key component of GEP.

The fourth element is the controlling parameter, which encompasses various parameters that influence the behavior and characteristics of the algorithm. Examples of controlling parameters include population size, mutation rate, and selection pressure. These parameters guide the evolution process and impact the exploration and exploitation balance within the population.

The fifth and final element is the terminating set, which is represented by letters (a, b, c, etc.). This set defines the variables or terminal symbols used in the expression trees. Terminal symbols are the building blocks of the expression trees and represent the inputs or variables in the mathematical equations.

The GEP algorithm starts by generating chromosomes randomly, which represent the genetic information. These chromosomes are then transformed into expression trees (ETs), and their fitness or health is evaluated using the fitness function. Superior individuals are selected for reproduction, and their genetic material is combined through processes like crossover and mutation to create new chromosomes in subsequent generations.

The iterative process continues until a satisfactory solution, determined by the termination condition, is found. During the decoding phase of GEP, each character in the gene is read from left to right and based on the grammar rules defined by the functional set and terminating set, gene maps are generated to construct the corresponding expression trees. These expression trees represent the mathematical equations that form the solutions sought by the GEP algorithm.

## 3 Results

### 3.1 The linear QSAR model of HM

**FIGURE 1:**
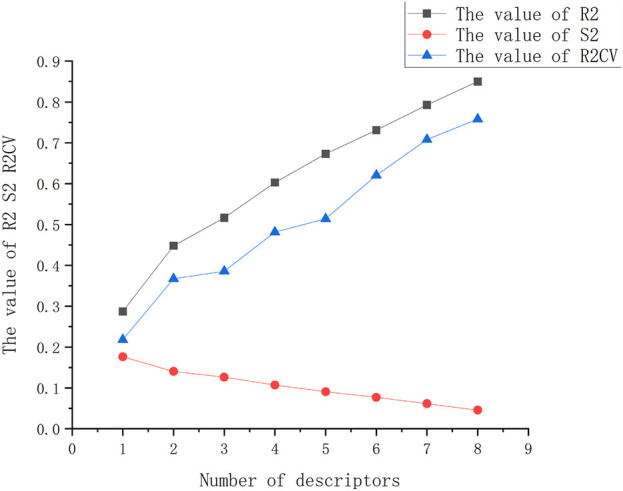
Influence of the number of descriptors on the *R* ^2^, R^2^_CV_, and S^2^. With number of descriptors increasing, *R* ^2^ and R^2^_CV_ increasing, at the same time, S^2^ decreasing. Combine *R* ^2^, R^2^_CV_, and S^2^, the four descriptors were chosen for future study.

**FIGURE 2:**
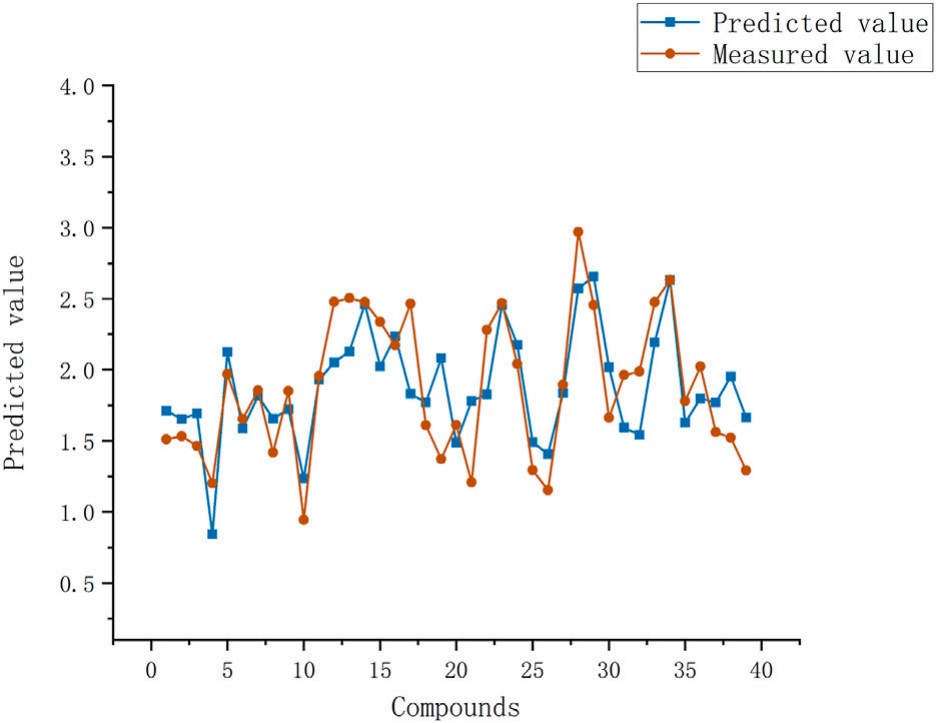
Plot of experimental and predicted log(IC_50_) values by heuristic method. Red line is measured values of all compounds, blue line is predicted values of HM of all compounds.

In order to determine the optimal number of descriptors that effectively describe the activity log (IC_50_) value of compounds, a heuristic method was employed. The process involved incrementally adding descriptors from 1 to 8 and evaluating the resulting statistical outcomes. It was observed that upon adding an additional descriptor, there were no significant changes in the statistical results, indicating that the appropriate number of descriptors had been reached. A total of 590 descriptors were calculated using the CODESSA project. Figure 1 depicts the relationship between the number of descriptors and the values of *R* ^2^ and R^2^_CV_. It can be observed that as the number of descriptors increased, both *R* ^2^ and R^2^_CV_ exhibited a steady upward trend. Additionally, the value of S^2^ decreased, indicating improved model performance. It is important to note that in QSAR studies, the number of molecular descriptors typically should not exceed 1/5 of the sample size. Based on the results obtained from the heuristic method, four descriptors with higher correlations were selected for further analysis. To ensure the independence of these descriptors, their pairwise correlation coefficients were examined and presented in Table 3. It was found that the coefficient between any two descriptors was lower than 0.8, indicating their independence and suitability for the model. The linear model constructed using these four descriptors is illustrated in Figure 2, providing a visual representation of the relationships between the descriptors and the activity log(IC_50_) values of the compounds.

**TABLE 3:** Correlation matrix of the descriptors in the model.

	MSA	ANRIFN	FFPQC	TM2RE
MSA	1.000	−0.072	0.474	−0.720
ANRIFN	−0.072	1.000	0.071	−0.104
FFPQC	0.474	0.071	1.000	−0.702
TM2RE	−0.720	−0.104	−0.702	1.000

With the increase in the number of descriptors, the performance metrics of the QSAR model display distinct patterns. Progressing from a single descriptor to four descriptors, the model witnesses incremental improvements in both crossvalidated *R* ^2^ and *R* ^2^ values, while simultaneously observing a decrease in both the F-statistic and s^2^ values. In the end, the utilization of four descriptors leads to a significant performance improvement, resulting in crossvalidated *R* ^2^ and *R* ^2^ values of 0.482 and 0.603, respectively. These values reflect the model’s enhanced ability to explain and predict the observed outcomes. Additionally, a consistent F-statistic of 10.80 underscores the robustness of the model, while the residual variance (s2) decreases to a notable 0.107. These outcomes underscore the effectiveness of the four selected descriptors in substantially enhancing the model’s predictive accuracy and explanatory power. The following equation highlights the representation of the optimal model based on the integration of these four descriptors.

$$\log\left({\text{IC}}_{50}\right)=-9.023-1.095e-03\text{MSA}+8.299\text{ANRIFN}+6.249e-02\text{FFPQC}+4.720TM2\text{RE}$$

### 3.2 The non-linear QSAR model of GEP

**FIGURE 3:**
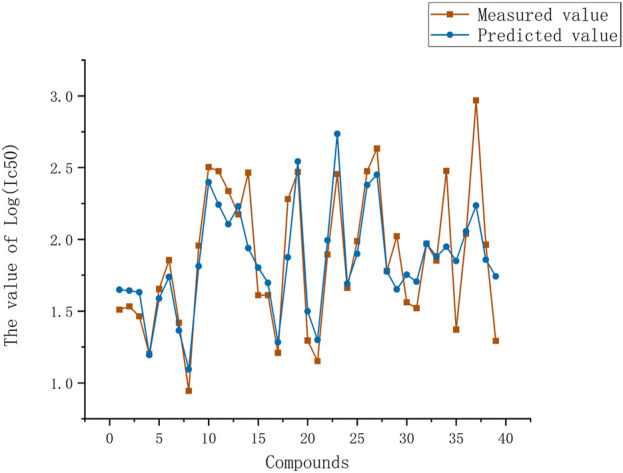
Predicted log(IC_50_) values versus experimental values by the gene expression programming. Red line is measured values of all compounds, blue line is predicted values of HM of all compounds. Most compounds have good predicted log(IC_50_) which agree with measured log(IC_50_) values.

The training set and test set were utilized as input in the automatic problem solver (APS, 3.0 2050, http://www.gepsoft.com/support/request.asp) program, utilizing the four selected descriptors as parameters. The APS program employed the training data to model the function and underwent multiple rounds of evolution to optimize the solution. This process led to the establishment of a nonlinear model for log(IC_50_), as illustrated in Figure 3. Using the GEP algorithm, the expression tree for the four parameters was computed. The expression tree was then transformed into a nonlinear mathematical equation, which can be expressed as follows:

$$\begin{array}{ll} \log\left({\text{IC}}_{50}\right) & =(\text{FFPQC}+(((1/\left(\text{MSA}\right))*\tan(\text{MSA}))/(1/(19.840)))))+(((1/((\tan\left(-2.954\right)-\text{FFPQC})))+0.153)\\ & +(\tan(((2\text{MSA})*(9.984/\left(-9.910+\text{TM}2\text{RE}\right)))+(1/((8.277/(\text{MSA}/(\left(\text{FFPQC}/\text{ANRIFN}\right)-(1/\left(\text{ANRIFN}\right)))))))\\ & +(1/((\text{MSA}*\tan((((\text{MSA}/d\left[2\right])*\text{ANRIFN})+(1/\left(\text{MSA}\right))))))) \end{array}$$

In the context of this GEP model, the best fitness and maximum fitness scores were determined to be 962.835 and 1000, respectively. The chosen functions for this model encompass addition, subtraction, multiplication, division, tangent tan, and inverse. The configuration entailed 100 chromosomes, each comprising 5 genes. The head size was set at 8, with a gene size of 26, and the chosen linking function was addition. Other parameters were maintained at their default values. The most optimal GEP model yielded *R* ^2^ values of 0.839 for the training set and 0.760 for the test set. Correspondingly, the associated S^2^ values for the training and test sets were calculated as 0.037 and 0.157, respectively.

### 3.3 Validation of compound with protein

**FIGURE 4:**
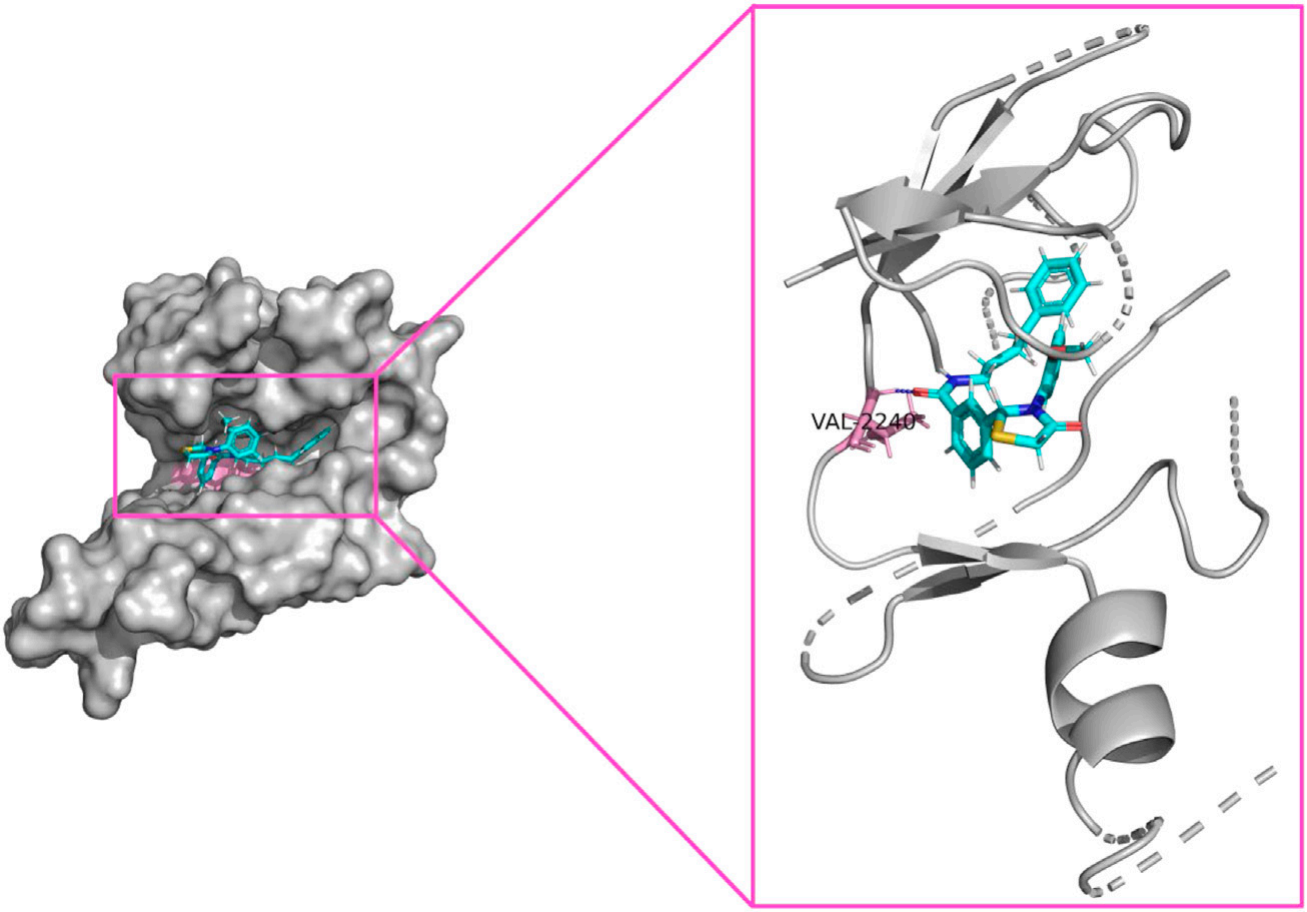
Compound 2j binding with osteosarcoma target (PDB code: 4JT5). The docking score (9.112), and an H-bonds of 2j compound with amino acid residue VAL-2240.

The 2j in Table 1 shows the lowest IC_50_ value, so this compound was selected for the future research and development. To explain relationship between the compound with osteosarcoma target (PDB code: 4JT5) and cancer, we have done docking study. In our study, we used suflex-dock method of software SYBYL-X 2.1.1 for docking work. Suflex-dock is a flexible docking program based on genetic algorithms. The 2j compound has higher dock score (9.112), and an H-bonds has found which is animo acid VAL-2240. Therefore, 2j compound can against osteosarcoma and cancer (Figure 4).

## 4 Discussion

During the evaluation of the QSAR model, commonly used indicators include the *R* ^2^ and S^2^. In the GEP model, the *R* ^2^ values for the training set are 0.839, and for the test set, they are 0.760. The corresponding S^2^ values for the training and test sets are 0.037 and 0.157, respectively. In comparison, the linear model constructed using the HM method yields *R* ^2^ values of 0.603 and S^2^ values of 0.107.

The GEP model demonstrates higher correlation coefficients for both the training and test sets compared to the HM model. Furthermore, the GEP model exhibits lower average errors in both the training and test sets compared to the HM model. When examining the curves of log (IC_50_) fitted by the two algorithms (Figures 2, 3), it becomes apparent that the GEP method provides superior fits for certain experimental values. The GEP model demonstrates a more pronounced ability to fit the data than the HM model.

Molecular surface area refers to the total surface area of a molecule, which includes both its external and internal surfaces. It is an important physical property that plays a role in various molecular processes, such as solubility, permeability, and interactions with other molecules. A negative coefficient for molecular surface area in our QSAR model implies that larger molecules, with a greater surface area, are associated with lower activity. This may be due to factors such as reduced solubility, decreased permeability, or limited interaction with biological targets (Tomalia et al., 1990).

The average nucleophilic reaction index for a nitrogen (N) atom (Hudson, 2010)is a descriptor that quantifies the nucleophilicity of a nitrogen atom in a molecule. It represents the tendency of the nitrogen atom to donate electrons in a chemical reaction. A positive coefficient associated with the average nucleophilic reaction index suggests a positive correlation between the nucleophilicity of the nitrogen atom and the activity of interest. This implies that higher nucleophilicity of the nitrogen atom is linked to decreased activity of the specific QSAR model.

FPSA-2 (Fractional PPSA) (Zhang et al., 2013) is a molecular descriptor used in quantitative structure-activity relationship studies. It represents the ratio of PPSA-2 (Partial Polar Surface Area of atom type 2) to TMSA (Total Molecular Surface Area) and is calculated using quantum-chemical calculations. The FPSA-2 descriptor can be utilized to assess the relationship between the fractional polar surface area and the activity or property of interest in a QSAR analysis. This positive correlation implies that as the fractional polar surface area contributed by atom type 2 increases relative to the total molecular surface area, the activity or property of interest tends to increase as well. It indicates that the presence and exposure of polar functional groups associated with atom type 2 may be associated with decrease activity or more favorable properties.

The “Total molecular 2-center resonance energy” is a descriptor that quantifies the degree of resonance stabilization within a molecule (Kobayashi et al., 2019). This descriptor reflects the energy associated with the redistribution of electron density resulting from the delocalization of π bonds or lone pairs within a molecule. In the context of a QSAR model, a positive coefficient associated with the “total molecular 2-center resonance energy” suggests a direct positive correlation between the resonance energy and the activity being studied. This implies that as the resonance energy of the molecule increases, the activity or property of interest tends to decrease. This outcome indicates that the presence of delocalized π bonds or lone pairs, which contribute to resonance stabilization, may lead to lower activity in the molecule. Resonance phenomena can influence various factors, including electronic distribution, charge density, and molecular stability, all of which can impact molecular interactions and reactivity. Therefore, understanding the role of resonance energy in a QSAR model can provide valuable insights into the relationships between molecular structure and activity.

Total 39 compounds were chosen from literature. ANRIFN is one of four descriptors which were selected in this work. All 39 structures were joined in descriptors finding. Descriptor has the highest efficient, so it has the great influence for activity. Based on the absolute values of the coefficients in the equation, the impact on the model can be ranked in the following order: ANRIFN > TM2RE > FFPQC > MSA. This ranking suggests that ANRIFN has the most significant effect on the model, followed by TM2RE, FFPQC, and MSA.

Both the HM and GEP algorithms have demonstrated strong predictive capabilities. However, upon conducting a comparative analysis of the results, it has been determined that GEP is better suited for the task at hand. The QSAR model developed using GEP incorporates nonlinear functions, allowing it to capture more complex relationships when compared to the linear functions generated by HM. While implementing the GEP algorithm may involve additional complexity due to genetic mutation and inversion of functional operations, it has yielded satisfactory results. Despite the challenges associated with GEP, it has proven to be capable of establishing a more accurate prediction model compared to HM. The statistical quality of the QSAR models, including their robustness and predictability, has been rigorously evaluated using various statistical methods. These results have yielded favorable results, indicating that the models exhibit desirable qualities in terms of accuracy, reliability, and their ability to predict the activity or properties of compounds. Detailed information and methodologies concerning the statistical analysis can be found in the referenced study.

## 5 Conclusion

Upon comparing the performance of HM and GEP, it becomes evident that GEP has constructed a significantly more accurate prediction model. The results show that HM achieved values of 0.603, while GEP achieved notably higher values of 0.839 and 0.760, respectively. These superior values indicate the advanced predictive capability of the GEP model. Through the application of the nonlinear GEP model, crucial elements that directly influence the IC_50_ value have been identified. These elements play a vital role in the selection of new molecules for further investigation. The study highlights the molecule’s significant impact on determining its IC_50_ value, allowing for potential modifications of the molecular structure to influence the IC_50_ value accordingly. In comparison to HM, the GEP model excels in terms of accuracy, enabling the identification of key elements that influence the IC_50_ value. This newfound understanding empowers researchers to make targeted modifications to molecular structures and thus manipulate the IC_50_ value. As a result, these findings contribute to the efficient utilization of resources and lead to a reduction in testing efforts, ultimately enhancing the drug discovery process.

## Data Availability

The original contributions presented in the study are included in the article/Supplementary material, further inquiries can be directed to the corresponding author.

## References

[B1] Almesned R. Azzam A. Z. Aldeheshi A. Amin T. M. (2023). Bidirectional intraoperative chemotherapy using cisplatin and ifosfamide for intraperitoneal mesothelioma in severe renal impairment: A case report. Am. J. Case Rep. 24, e938192. 10.12659/AJCR.938192 36964641 PMC10052470

[B2] Azadian R. Mohammadalipour A. Memarzadeh M. R. Hashemnia M. Aarabi M. H. (2023). Examining hepatoprotective effects of astaxanthin against methotrexate-induced hepatotoxicity in rats through modulation of Nrf2/HO-1 pathway genes[J]. Naunyn‐Schmiedeb. Arch. Pharmacol., 1–10. 10.1007/s00210-023-02581-8 37450013

[B3] Deng Y. Pi R. Niu L. Zhao Y. Ni D. Song L. (2022). Novel 2-phenyl-3-(Pyridin-2-yl) thiazolidin-4-one derivatives as potent inhibitors for proliferation of osteosarcoma cells *in vitro* and *in vivo* . Eur. J. Med. Chem. 228, 114010. 10.1016/j.ejmech.2021.114010 34861640

[B4] Esparragosa Vazquez I. Ndiaye M. Di Stefano A. L. Younan N. Larrieu-Ciron D. Seyve A. (2023). T2-Fluid-attenuated inversion recovery (FLAIR) pseudoprogression in patients with anaplastic oligodendrogliomas treated with procarbazine, lomustine and vincristine (PCV) chemotherapy alone. Eur. J. Neurol. 30, 2879–2883. 10.1111/ene.15873 37204066

[B5] Feng X. J. Yan N. Wang Y. Mei P. Chen W. Lu L. (2023). Corrosion inhibition studies of 8-hydroxyquinoline derivatives for N80 steel in a 1.0 M HCl solution: Experimental, computational chemistry, and quantitative structure-activity relationship studies. Langmuir 39, 519–532. 10.1021/acs.langmuir.2c02807 36562562

[B6] Froimowitz M. (1993). HyperChem: A software package for computational chemistry and molecular modeling. Biotechniques 14, 1010–1013. 8333944

[B7] Ghashghaeinia M. Koberle M. Mrowietz U. Bernhardt I. (2019). Proliferating tumor cells mimick glucose metabolism of mature human erythrocytes. Cell. Cycle 18, 1316–1334. 10.1080/15384101.2019.1618125 31154896 PMC6592250

[B8] Gieseking R. L. M. (2021). A new release of MOPAC incorporating the INDO/S semiempirical model with CI excited states. J. Comput. Chem. 42, 365–378. 10.1002/jcc.26455 33227163

[B9] Hua H. Zhao Q. Xia J. Dai Q. L. Bai S. R. Wang X. B. (2023). Peficitinib ameliorates doxorubicin-induced cardiotoxicity by suppressing cellular senescence and enhances its antitumor activity. Int. Immunopharmacol. 122, 110630. 10.1016/j.intimp.2023.110630 37451017

[B10] Hudson H. (2010). Nucleophilic reactions of phosphines, 385–471.

[B11] Ipek S. Degtyarev V. V. Guneyisi E. M. Mansouri I. (2022). GEP-based models for estimating the elastic shear buckling and ultimate loads of cold-formed steel channels with staggered slotted web perforations in shear. Structures 46, 186–200. 10.1016/j.istruc.2022.10.060

[B12] Karaduman G. Kelleci Celik F. (2023). 2D-Quantitative structure-activity relationship modeling for risk assessment of pharmacotherapy applied during pregnancy. J. Appl. Toxicol. 10.1002/jat.4475 37082782

[B13] Katritzky A. R. Dobchev D. A. Tulp I. Karelson M. Carlson D. A. (2006). QSAR study of mosquito repellents using Codessa Pro. Bioorg Med. Chem. Lett. 16, 2306–2311. 10.1016/j.bmcl.2005.11.113 16488605

[B14] Kobayashi H. Picard L.-P. Schönegge A.-M. Bouvier M. (2019). Bioluminescence resonance energy transfer–based imaging of protein–protein interactions in living cells. Nat. Protoc. 14, 1084–1107. 10.1038/s41596-019-0129-7 30911173

[B15] Kucan D. Orsolic N. Odeh D. Ramic S. Jakopovic B. Knezevic J. (2023). The role of hyperthermia in potentiation of anti-angiogenic effect of cisplatin and resveratrol in mice bearing solid form of ehrlich ascites tumour. Int. J. Mol. Sci. 24, 11073. 10.3390/ijms241311073 37446252 PMC10341868

[B16] Lakshmi P. J. Apaza R. A. Alkhayyat A. Marhoon H. A. Alameri A. A. (2022). Hybrid wavelet-gene expression programming and wavelet-support vector machine models for rainfall-runoff modeling. Water Sci. Technol. 86, 3205–3222. 10.2166/wst.2022.400 36579879

[B17] Tomalia D. A. Naylor A. M. Goddard W. A. Iii (1990). Starburst dendrimers: Molecular-level control of size, shape, surface chemistry, topology, and flexibility from atoms to macroscopic matter. Angewandte Chemie Int. Ed. Engl. 29, 138–175. 10.1002/anie.199001381

[B18] Wang D. Zhu Q. Zhang X. Zhang L. He Q. Yang B. (2010). Hypoxia promotes etoposide (VP-16) resistance in neuroblastoma CHP126 cells. Pharmazie 65, 51–56. 20187579

[B19] Yang L. Long Y. Xiao S. (2023). Osteosarcoma-associated immune genes as potential immunotherapy and prognosis biomarkers. Biochem. Genet. 10.1007/s10528-023-10444-3 37452172

[B20] Zhang Y. Li S. Kong X. (2013). Relationship between antimold activity and molecular structure of cinnamaldehyde analogues. Bioorg. Med. Chem. Lett. 23, 1358–1364. 10.1016/j.bmcl.2012.12.085 23374870

[B21] Zhang Y. Zhao X. Gao C. Lin L. Y. Li Y. (2023). Treatment outcome analysis of bevacizumab combined with cyclophosphamide and oxaliplatin in advanced pseudomyxoma peritonei. World J. Gastrointest. Surg. 15, 1149–1158. 10.4240/wjgs.v15.i6.1149 37405093 PMC10315110

