# Supplementary figures and images for: A novel non-linear approach for establishing a QSAR model of a class of 2-Phenyl-3-(pyridin-2-yl) thiazolidin-4-one derivatives
Source: Front Pharmacol. 2023 Sep 27;14:1263933. doi: 10.3389/fphar.2023.1263933 (PMC10565811; doi:10.3389/fphar.2023.1263933)

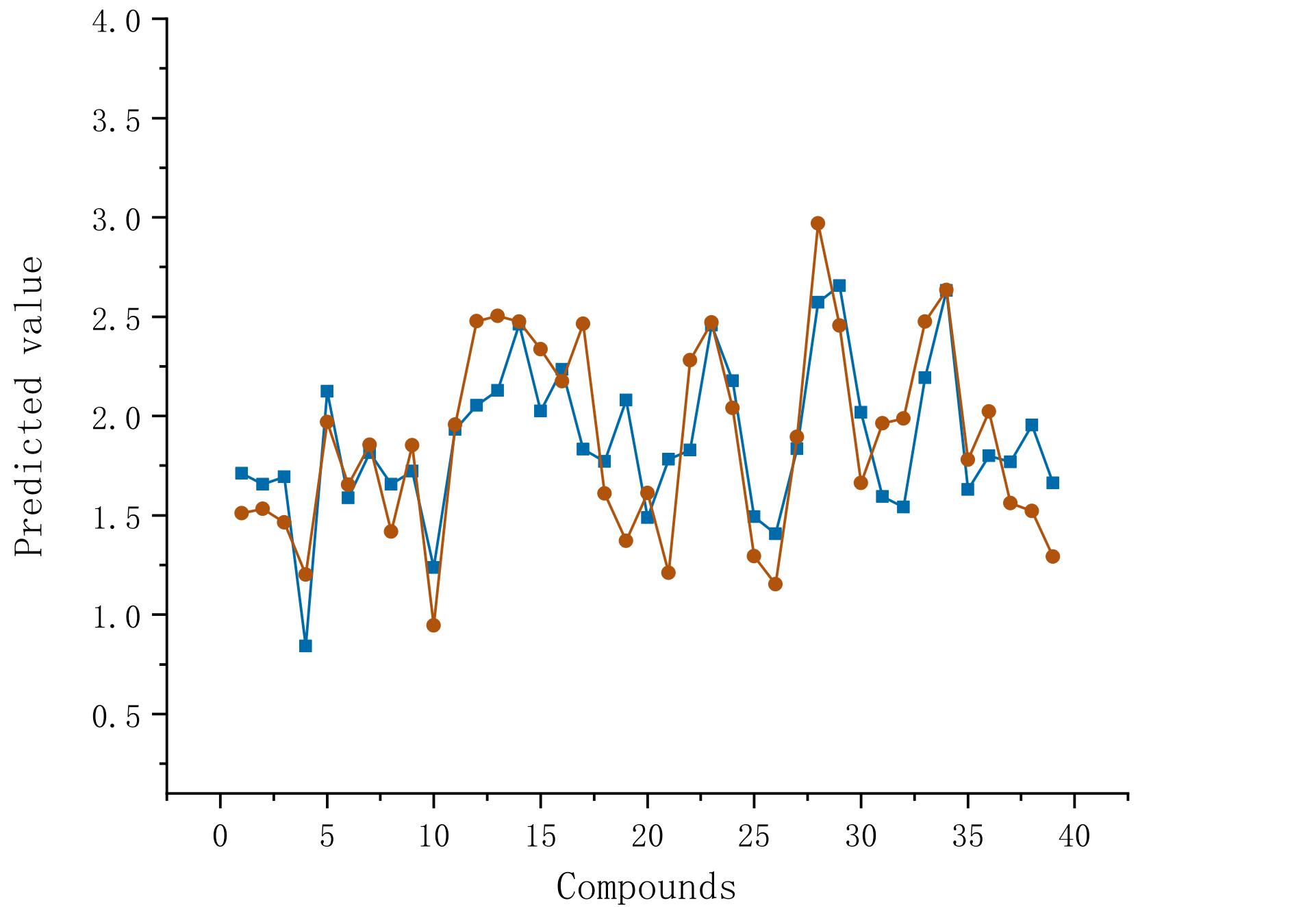

Supplement: Supplementary file 1 [file DataSheet2.PDF]

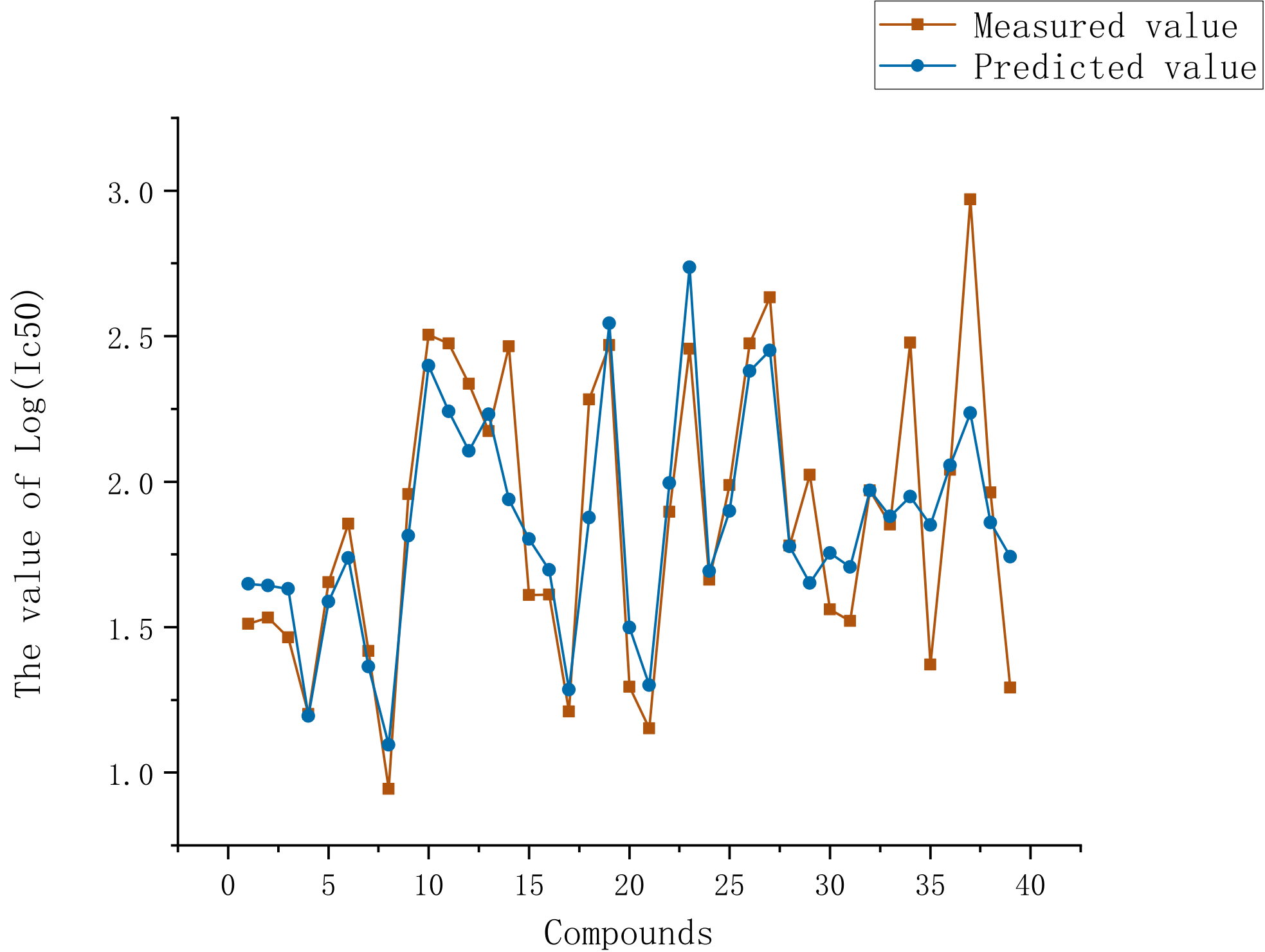

Supplement: Supplementary file 4 [file DataSheet3.PDF]

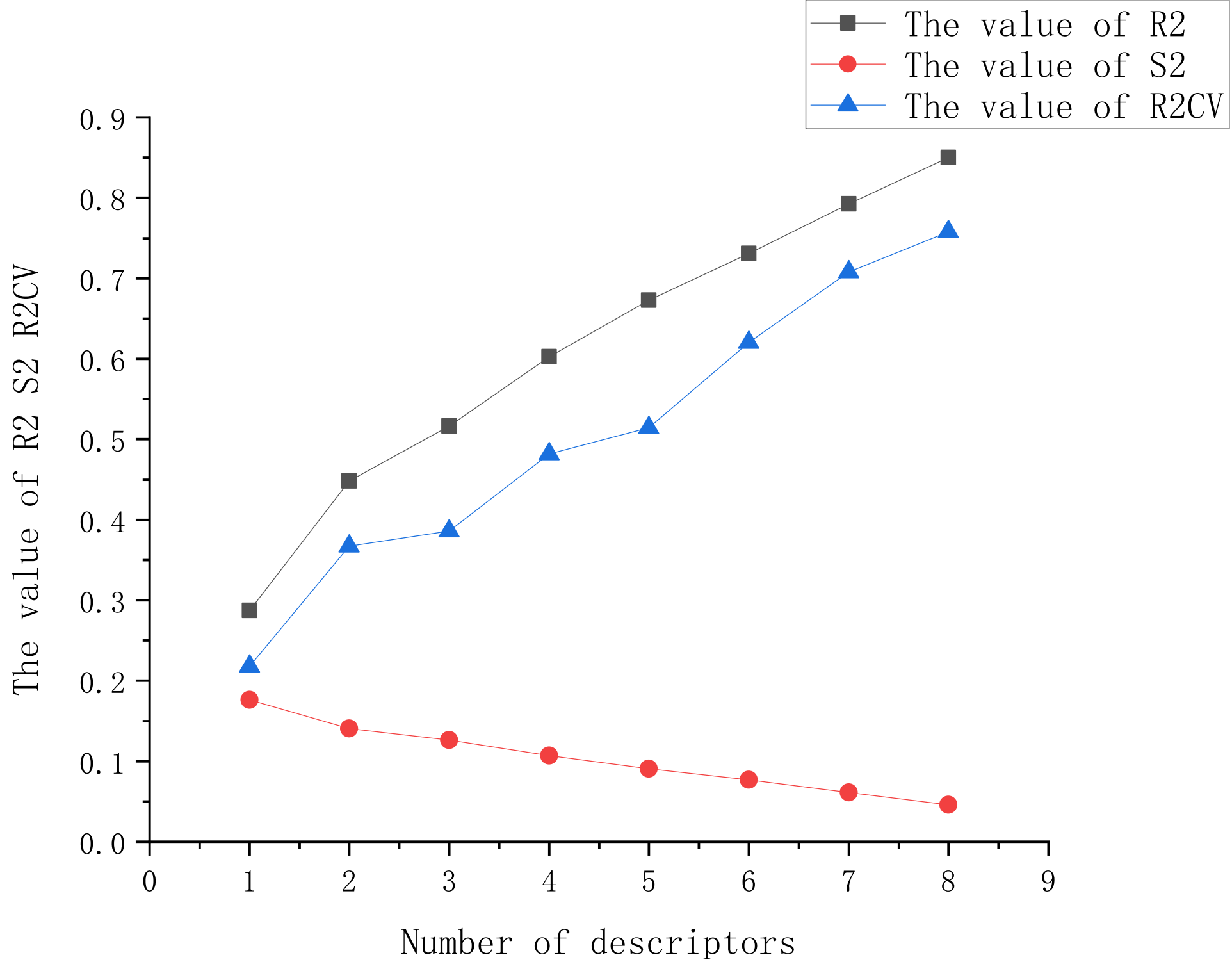

Supplement: Supplementary file 5 [file DataSheet1.PDF]
